# Phenotypical and Molecular Characterization of *Acinetobacter baumannii* Isolated from Hospitalized Patients During the COVID-19 Pandemic in Brazil

**DOI:** 10.3390/life15040623

**Published:** 2025-04-08

**Authors:** Paula Araujo de Souza, Milena Cristina Nunes dos Santos, Rebeca Vitória da Silva Lage de Miranda, Luciana Veloso da Costa, Raphael Paiva Paschoal da Silva, Cátia Aparecida Chaia de Miranda, Greice Maria Silva da Conceição, Stephen James Forsythe, Maria Helena Simões Villas Bôas, Marcelo Luiz Lima Brandão

**Affiliations:** 1 Laboratory of Microbiology of Food and Sanitizes, INCQS/Fiocruz, Rio de Janeiro 21040-900, Brazil; maria.villas@fiocruz.br; 2 Laboratory of Microbiological Control, Bio-Manguinhos/Fiocruz, Rio de Janeiro 21040-900, Brazil; milena.santos@bio.fiocruz.br (M.C.N.d.S.); rebeca.lage@bio.fiocruz.br (R.V.d.S.L.d.M.); luciana.costa@bio.fiocruz.br (L.V.d.C.); marcelo.brandao@bio.fiocruz.br (M.L.L.B.); 3 Hospital de Força Aérea do Galeão, Força Área Brasileira, Rio de Janeiro 21941-353, Brazil; raphaelpasc@gmail.com; 4 Interdisciplinary Medical Research Laboratory, IOC/Fiocruz, Rio de Janeiro 21040-900, Brazil; catia.chaia@ioc.fiocruz.br; 5 Analytical Indicators and Data Systems Section, Bio-Manguinhos/Fiocruz, Rio de Janeiro 21040-900, Brazil; greice.conceicao@bio.fiocruz.br; 6 Foodmicrobe.com Ltd., Adams Hill, Keyworth, Nottingham NG12 5GY, UK; sforsythe4j@gmail.com

**Keywords:** *A. baumannii*, COVID-19, antimicrobial resistant, biofilm, FTIR, MALDI TOF/MS, 16S rRNA sequencing, typing, epidemiology, healthcare-associated infections

## Abstract

The goal of the present study was to analyze *Acinetobacter baumannii* strains isolated from hospitalized patients in the period of the COVID-19 pandemic by phenotypic and molecular methods and evaluate their antimicrobial resistance patterns and biofilm production. Forty-seven strains were isolated in 2021–2022 from a hospital in Brazil, and were identified by VITEK^®^2, MALDI-TOF/MS (VITEK MS^®^ and MALDI Biotyper^®^), and 16S rRNA sequencing. Fourier-transform infrared (FTIR) spectroscopy was applied for typing and antimicrobial susceptibility testing (AST). In addition, biofilm formation and disinfectant tolerance tests were used. All methods accurately identified all the *A. baumannii* strains. FTIR typing identified 23 different profiles and 11 clusters, as well as differentiated between the strains from patients with and without COVID-19. Most strains exhibited resistance to the drugs tested, 91.5% of the strains were classified as XDR, 6.4% of the strains were MDR and only 1 strain was classified as non-MDR. Over half of the strains (n = 27, 57.4%) produced biofilms on polystyrene. Sodium hypochlorite (1.0%/15 min) was the best option for effective disinfection. Overall, this study will lay the foundation for further research on effective cleaning protocols for the eradication of *A. baumannii* biofilms, as well as the use of FTIR for pathogen surveillance in healthcare settings.

## 1. Introduction

*Acinetobacter baumannii* is considered to be a crucial threat to public health, especially owing to its high potential to obtain multidrug resistance phenotypes, enabling the emergence of multidrug-resistant (MDR), extensively drug-resistant (XDR) or even pandrug-resistant (PDR) strains [1,2,3,4,5]. According to the Food and Agriculture Organization of the United Nations (FAO) [6], it is estimated that by 2050, antimicrobial resistance could cause about 10 million deaths annually, resulting in a reduction of 2 to 3.5% in global Gross Domestic Product (GDP), a loss equivalent to USD 100 trillion.

*A. baumannii* is one of the most relevant opportunistic bacteria associated with healthcare-associated infections (HAIs) [7] and is listed in the critical group in the Bacterial Priority Pathogens List published by the WHO [8]. One of the main reasons why this microorganism is so hard to eradicate in the hospital environment is its ability to form a biofilm on medical equipment [9,10,11].

The typing of microorganisms in hospitals often relies on overpriced and laborious techniques that are restricted to retrospective research [12]. Therefore, the implementation of quick and accurate typing techniques into routine microbiology laboratories would accelerate the inspections and improve a hospital’s contamination control strategy and the traceability of microorganisms [13]. The Fourier-transform infrared (FTIR) spectroscopy is a technique that involves typing microorganisms within three hours [14]. This technique has been a successful tool for the surveillance of *A. baumannii* and for the detection of its clonal spreading in hospital outbreaks [13,14].

*A. baumannii* is often the cause of many clinically important hospital infections, such as pneumonia associated with the use of mechanical ventilation (VAP), septicemia, urinary tract infections and post-neurosurgical meningitis [15]. VAP has been considered a challenge due to its high mortality incidence [16]. VAP caused by *A. baumannii* was widely reported in patients with SARS-CoV-2 during the COVID-19 pandemic [5,17,18,19,20,21]. This could be attributed to the ability of this microorganism to produce biofilms on medical devices used for endotracheal intubation leading to an increase in *A. baumannii*–SARS-CoV-2 co-infections [17].

This study aimed to analyze *A. baumannii* strains isolated from hospitalized patients in the period of the COVID-19 pandemic using phenotypic and molecular techniques and evaluate its virulence factors.

## 2. Materials and Methods

### 2.1. Strains and Cultivation Conditions

Forty-seven isolates identified as *A. baumannii* complex using VITEK^®^2 (bioMérieux, Craponne, France), with a confidence of >99%, were isolated between June 2021 and March 2022 from a hospital in Rio de Janeiro State, Brazil. They were isolated from 33 patients, and from several sources: tracheal secretion (n = 16), blood (n = 11), rectal swab (n = 10), urine (n = 6), and tracheal swab (n = 4) (Table 1). The strains were isolated from patients with COVID-19 and without COVID-19 coinfection, and the COVID-19 tests were performed using AllplexTM 2019-nCoV Assay (version 2.1).

The strains were maintained at −70 °C in Brain Heart Infusion broth (BHI, Merck, Darmstadt, Germany) containing 20% glycerol (Merck, Darmstadt, Germany). Strains were deposited at the Coleção de Bactérias do Ambiente e Saúde (CBAS)/Fundação Oswaldo Cruz (Fiocruz). CBAS is affiliated with the World Federation for Culture Collections (WFCC) and registered as the World Data Centre for Microorganisms (WDCM) 958. The *Pseudomonas aeruginosa* ATCC 27853 strain was used as the positive control in the biofilm assays.

### 2.2. Identification by Matrix-Assisted Laser Desorption Ionization–Time of Flight Mass Spectrometry (MALDI-TOF MS) Proteomic Characterization

The strains were seeded on Sheep Blood Agar (SBA) (BioCen do Brasil, São Paulo, Brazil) at 32.5 ± 2.5 °C/48 h. MALDI Biotyper^®^ (Bruker Daltonics, Bremen, Germany) and VITEK MS^®^ systems (bioMérieux, Craponne, France) were used according to the manufacturer’s instructions to identify all isolates.

For the MALDI Biotyper^®^, a portion of the colony was suspended in 300 μL of sterile water and after vigorous homogenization, 900 μL of absolute ethanol (Merck KGaA, Darmstadt, Germany) was added. After another round of vigorous homogenization, the sample was centrifugated for 2 min. The supernatant was discarded and the centrifugation was performed again to ensure its complete removal. The pellet was dried at room temperature for five minutes with the tube cap open. Next, 50 μL of 70% formic acid was added and, after homogenization, 50 μL of acetonitrile was added to the pellet. After further centrifugation under the same conditions, one microlitre of supernatant was applied to the spot of the slide. After drying, 1 μL of Bruker HCCA was added to the spots. The calibration was performed using BTS spot with Flex Control v.3.4, according to manufacturer’s instructions. The results were evaluated using Bruker Species Entry List MBT Compass Library Revision K (2022), and the scores ≥ 2.00 were considered correctly identified at the species level with high confidence. For VITEK MS^®^, a portion of the colony was applied to the slide together with 1.0 µL of formic acid 70% and, after drying, 1.0 µL of al-pha-cyano-4-hydroxycinnamic acid matrix solution. The results were evaluated using the Saramis Premium (version 4.0.0.14) program, and the microorganism was considered correctly identified at the species level for results ≥ 75%.

### 2.3. Identification by 16S rRNA Gene Sequencing Analysis

The identification of the strains was accomplished by 16S rRNA gene sequencing using the MicroSEQ™ Full Gene 16S rDNA kit (Thermo Fisher Scientific, Waltham, MA, USA), according to the manufacturer’s instructions. DNA Star LaserGene SeqMan software v.7.0.0 was used for sequence processing, and the results were acquired from the following website: https://www.ezbiocloud.net/ (database update: 7 July 2021; last access: 23 February 2023). Only isolates with a species identification percentage ≥ 98.7% were considered valid [22]. All sequences were deposited at https://www.ncbi.nlm.nih.gov/ and the access numbers are provided in Table A1. The sequences of the strains *A. baumannii* ATCC 19606 (access number ACQB01000091), *A. haemolyticus* CIP64.3 (access number APQQ01000002), *A. halotolerans* R160 (access number KT032155), *A. seifertii* NIPH973 (access number KB851199), *A. nosocomialis* NIPH2119 (access number APOP01000014) and *Moraxella lacunata* NBRC 102154 (access number BCUK01000202) were included in the phylogenetic analysis. A neighbor-joining phylogenetic tree was built based on multiple alignments of nearly complete 16S rRNA gene sequences using the ClustalW algorithm with the software MEGA 11 by employing the Kimura 2-parameter model with 1000 bootstrap replicates [23].

### 2.4. Typing by FTIR Spectroscopy Analysis

Strains were seeded on TSA at 37 ± 1 °C/24 h. Samples were prepared in a room where the temperature was 22 ± 2 °C. A small portion of each colony was mixed well with ethanol 70% (50 μL), and 50 μL distilled water was added and mixed again. Fifteen microliters were dispensed into 3 spots onto a 96-well silicon microplate. The plate dried at room temperature and was introduced to the IR Biotyper^®^ (Bruker Optics-Daltonics GmbH, Bremen, Germany). IRTS1 and IRTS2 standards were spotted in duplicate. The raw data were used to create a dendrogram to cluster the separation spectrum. A cut-off value was calculated by the program OPUS v.7.5 (Bruker Optics-Daltonics GmbH).

### 2.5. Evaluation of Antimicrobial Susceptibility Profile

*A. baumannii* strains were analyzed for antimicrobial susceptibility profiles using the Kirby–Bauer method. The antimicrobials tested included meropenem (MEM), imipenem (IPM), sulfamethoxazole–trimethoprim (SXT), piperacillin–tazobactam (TZP), amikacin (AMI), gentamicin (GEN), ciprofloxacin (CIP), ceftazidime (CAZ), cefoxitin (FOX), cefepime (CEF), ceftriaxone (CRO), tigecycline (TIG) and ampicillin–sulbactam (AMS). The results were interpreted following the Clinical and Laboratory Standards Institute [24].

Isolates were classified as multi-drug resistant (MDR), extensively drug-resistant (XDP) or pandrug-resistant (PDR) according to Magiorakos et al. [1].

### 2.6. Evaluation of Biofilm Formation and Its Tolerance to Disinfectants

The strains were tested for biofilm formation on the polystyrene surface of 96 well-plates (Falcon^®^, Jersey, NJ, USA) as described by Vasconcellos et al. [25]. The experiment was repeated in independent experiments (n = 3) for each strain in triplicate. The strains were grown on tubes containing 10 mL of BHI using sterilized inoculating loops and incubated at 37 °C/24 h with shaking. The negative control tube contained BHI only. *P. aeruginosa* ATCC 27853 was used as a control for biofilm production. Three wells of sterile 96-well polystyrene plates were filled with 200 µL of bacterial suspension each. The plates were covered and incubated at 22.5 ± 2.5 °C and 37 ± 2 °C for 48 h. Then, the contents of each plate were removed and rinsed 6x with water and air-dried for 45 min. After drying, the plates were stained with 200 µL of 0.41% crystal violet for 45 min. Then, the content of each plate was removed and rinsed 6x with running tap water and air-dried for 45 min. The crystal violet bound to the biofilm was dissolved with 200 µL of 96% ethanol (Merck, Darmstadt, Germany) for 10 min. One hundred and fifty microliters of each well was added to a new sterile 96-well polystyrene plate, and absorbance was determined at 600 nm by a microplate reader (Biomérieux, Reader 270, France). Strains were categorized, based on the cut-off optical density (ODC) compared with OD of the negative control, as non-adherent (-), weakly adherent (+), moderately adherent (++), and strongly adherent (+++) [26]. The final classification was the medium of all results.

Tolerance to disinfectants was evaluated for isolates classified as moderately or strongly adherent. The disinfectants tested were as follows: alcohol 70% (15 min) (Merck, Darmstadt, Germany), sodium hypochlorite (0.1% and 0.5%, 15 min) (Brasquímica, Belo Horizonte, Brazil), disinfectant based on the synergistic association between ammonium quaternary 5th generation and stabilized polymeric biguanide (10 min) (Mirax BG diluted 1:200 and 1:300 Hortolândia, Brazil), and peracetic acid 0.5% (10 min) (Divosan Forte VT6, Diversey^®^, Peróxidos do Brasil Ltd., Curitiba, Brazil) at the temperature of 37 ± 2 °C. Wilcoxon signed ranks test (R Core Team v.4.2.0, Vienna, Austria) was applied to verify the differences in biofilm formation and its tolerance to disinfectants. *p*-values < 0.05 were considered significant.

## 3. Results

### 3.1. Identification and Typing

**Figure 1:**
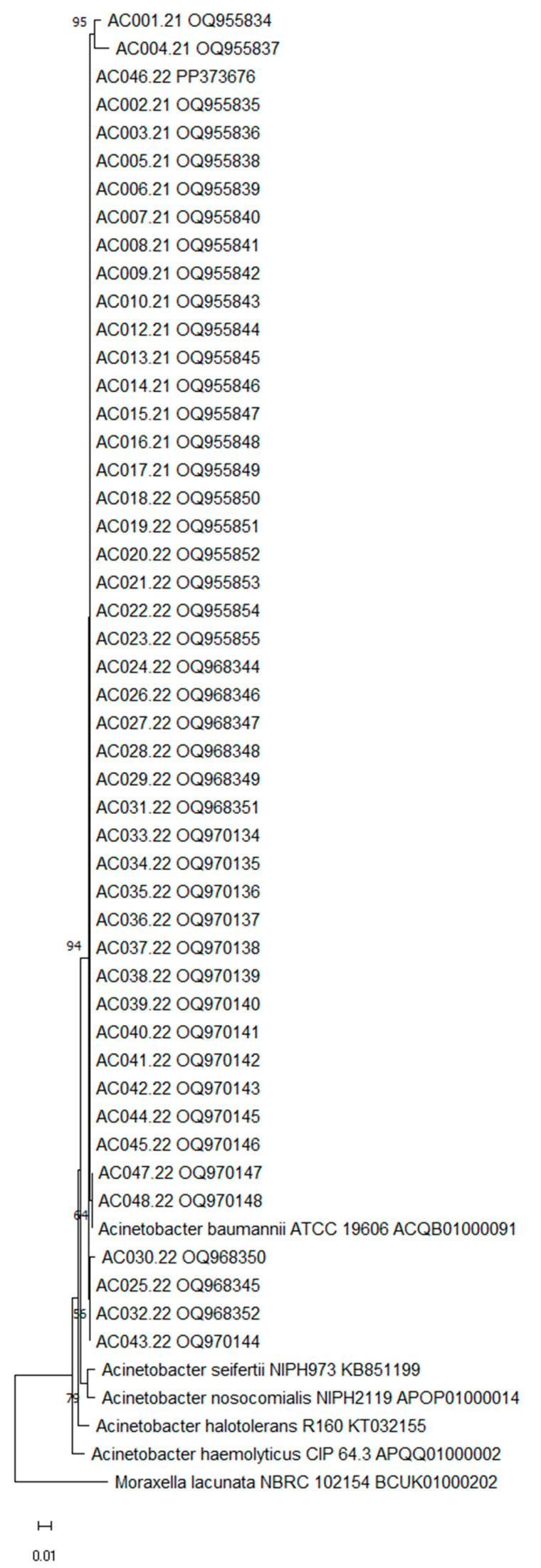
Neighbor-joining tree based on partial 16S rRNA gene sequences (549 bp) showing the phylogenetic position of the *Acinetobacter baumannii* strains evaluated in the present study (n = 47). The numbers at the nodes indicate the percentage of 1000 bootstrap replicates; only values > 50% are shown. *A. seifertii*, *A. nosocomialis*, *A. halotolerans*, *A. haemolyticus* and *Moraxella lacunata* was used as an outgroup. The scale bar represents 0.01 substitutions per nucleotide position. GenBank accession number is given.

VITEK MS^®^ and MALDI Biotyper^®^ identified all isolates as *A. baumannii* with ≥80.1% confidence and a score of ≥2.02, respectively (Table 1). Similarly, 16S rRNA gene sequencing identified the strains as *A. baumannii* with a similarity ≥90.90% (Table A1). The neighbor-joining tree is presented in Figure 1.

**Figure 2:**
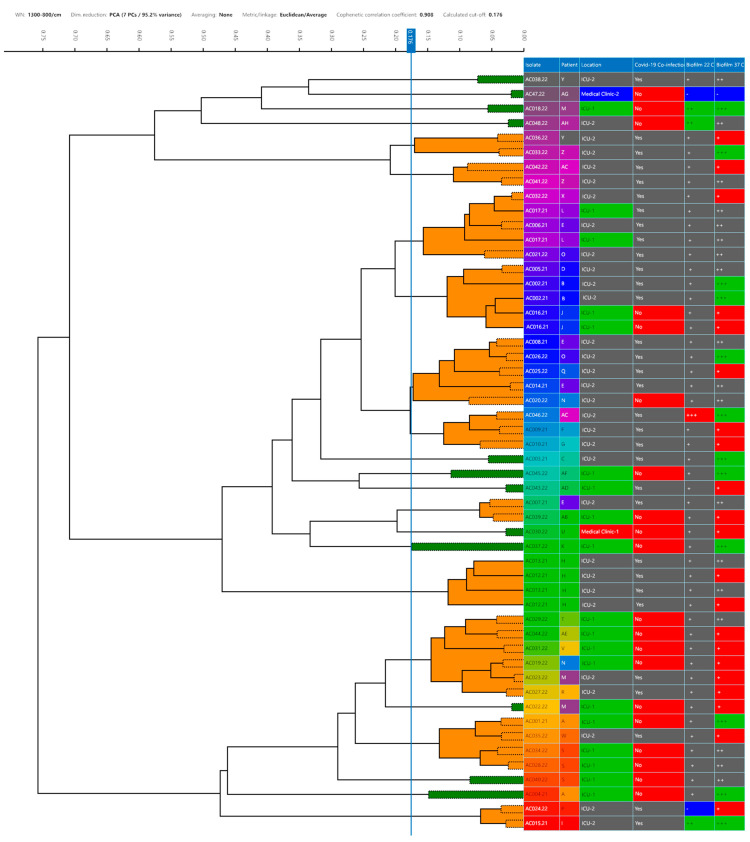
Dendrograms obtained by clustering FTIR spectra for *Acinetobacter baumannii* strains (n = 47). The vertical line represents the cut-off value. Green spectra indicate that the strain was a singleton, and orange spectra indicate a cluster formation. Different colors are used to differentiate: source; COVID-19 co-infection (yes or no); location and biofilm formation on stainless-steel surfaces (+ = positive; ‘-’= negative) and polystyrene (‘-‘ = non-adherent; + = weakly adherent; ++ = moderately adherent; and +++ = strongly adherent).

According to FTIR spectroscopy, the strains showed 23 distinct profiles and formed 11 clusters with a 0.176 cut-off (Figure 2). The FTIR profile 18 formed the biggest cluster with six strains (AC019, AC023, AC027, AC029, AC031 and AC044) followed by profile 9 with five strains (AC008, AC014, AC020, AC025 and AC026) and profiles 7 (AC006, AC017, AC021 and AC032) and 20 (AC001, AC028, AC034 and AC035) with four strains each.

Both profiles 8 (AC002, AC005 and AC016) and 10 (AC009, AC010 and AC046) grouped three strains together. Finally, the FTIR profiles 5 (AC033 and AC036), 6 (AC041 and AC042), 14 (AC007 and AC039), 17 (AC012 and AC013) and 23 (AC015 and AC024) formed the smallest groups, clustering two strains each.

The FT-IR was also able to cluster most of the strains that were isolated from the same patient. Profile 9 grouped two strains (AC008 and AC014) from patient E. The same was observed in profile 17, where the strains AC012 and AC013, both from patient H, were clustered together. The strains AC028 and AC034, isolated from patient S, were clustered in FT-IR profile 20. Even though strain AC040 was also from patient S and was not clustered in profile 20, it was aligned side by side with the other strains from the same patient at the dendrogram. Finally, the strains AC033 and AC044, isolated from patient Z, were not grouped in the same cluster; however, they were grouped in profiles very close to each other, profiles 5 and 6, respectively.

### 3.2. Antimicrobial Susceptibility Profile

Regarding the antimicrobial susceptibility profile, the strains showed the same percentage of resistance (95.8%) and susceptibility (4.2%) to TZP, CAZ, CEF and MEM. The same occurred with SXT and GEN, where 93.6% of the strains were resistant and 6.4% were susceptible.

The same percentage of resistance (97.9%) was also observed to IPM, FOX and CIP, while 4.2% were susceptible to IPM and intermediately resistant to FOX and CIP. Regarding the TIG, 21.3% of the strains were resistant, while 36.2% were intermediate resistant, and finally, 42.5% showed susceptibility to this antibiotic.

The same percentage of resistance (87.3%) was observed for CRO, AMI and AMS. Concerning AMS, 4.2% of the strains were intermediately resistant and 8.5% were susceptible. For CRO, 10.6% were intermediately resistant and only 2.1% were susceptible. Finally, for AMI, 12.8% were susceptible.

Detailed information on the results of the antimicrobial susceptibility profile and Magiorakos et al. [1] classification can be observed in Table A2. Forty-three (91.5%) strains were classified as XDR, three (6.4%) strains were MDR and only the strain AC015 was classified as non-MDR. The antimicrobial TIG is not on Magiorakos et al. [1] list since it is a new drug, a glycylcycline class antibiotic. Nevertheless, it was used in the classification in the present study.

### 3.3. Biofilm Formation and Its Tolerance to Disinfectants

**Figure 3:**
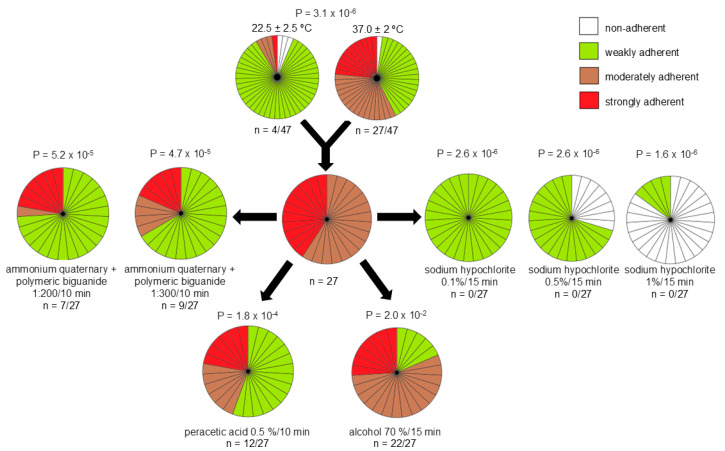
Biofilm formation of *Acinetobacter baumannii* strains (n = 47) and assessment of biofilm sensitivity to disinfectants. Color legend: white—non-adherent; green—weak adherent; brown—moderate adherent; red—strong adherent. *p*-values are presented.

The biofilm production results can be found in Figure 3. A significant statistical dissimilarity between the temperatures of 22.5 ± 2.5 °C and 37 ± 2 °C was noticed (*p* = 3.2 × 10^−6^). At the temperature of 22.5 ± 2.5 °C, 6.4% (n = 3) of the strains formed moderately adherent biofilms, while one strain formed strongly adherent biofilms. At 37 ± 2 °C, 34.0% (n = 16) of the strains formed moderately adherent biofilmx and 23.4% (n = 11) of the strains formed strongly adherent biofilms.

The 27 (57.4%) strains categorized as moderately or strongly adherent were selected for the biofilm disinfectant tolerance test. Peracetic acid 0.5% was able to reduce the biofilm of fifteen (55.5%) strains to weakly adherent, while the alcohol 70% only reduced the biofilm of five (18.5%) strains. The ammonium quaternary 5th generation stabilized polymeric biguanide diluted 1:300 was able to reduce 18 (66.7%) strains to weakly adherent, while at 1:200 dilution, it reduced the biofilm formation of 20 (74.1%) strains. Exposure to 0.1% sodium hypochlorite for 15 min reduced all the strains to weakly adherent and the concentration of 0.5% reduced the biofilm of eight (29.6%) strains to non-adherent, while the concentration of 1.0% reduced twenty-three (85.2%) strains to non-adherent. Significant statistical values were observed in all disinfectant tolerance tests (*p* ≤ 1.8 × 10^−4^), with the exception of alcohol at 70% (*p* = 0.02).

## 4. Discussion

*A. baumannii* is one of the most prevalent pathogens in hospital-acquired nosocomial infections, and therefore its accurate identification is essential [3,15,17]. Correct bacteria identification is fundamental for establishing efficient disinfection methods, infection control strategies and for the correct use of antibiotics [2]. In this study, all three methods used for identification (VITEK MS^®^, MALDI Biotyper^®^ and 16S rRNA sequencing) were able to identify all 47 strains to the *Acinetobacter* species level. Both MALDI TOF/MS systems used possess *A. baumannii* species in their libraries, and extraction protocols were sufficient to identify the strains with high confidence. These results were similar to previous reports using MALDI-TOF MS and 16S rRNA sequencing for *A. baumannii* identification [25,27]. Toh et al. [28] reported that MALDI-TOF MS was able to differentiate and identify 100% (n = 47) of the *A. baumannii* strains even from those that are closely related, such as *A. pittii* and *A. calcoaceticus*. Vasconcellos et al. [25] also reported that MALDI-TOF MS was sufficient to identify *A. baumannii* strains (n = 4) isolated from a pharmaceutical facility; however, it was not enough to differentiate other species of the *Acinetobacter calcoaceticus–baumannii* complex.

The FT-IR resulted in 23 distinct profiles and 11 clusters where the IR profiles 5, 6, 7, 8, 9, 10, 14, 17 and 23 clustered the strains related to co-infections with SARS-CoV-2, except for AC016 and AC020 (Figure 2). It was not observed that there was a relation between antimicrobial resistance and the FT-IR clusters, since only N-MDR strain was found (AC15), clustered with AC24, an MDR strain (Table A2). Regarding biofilm production, stronger biofilm producers’ strains were found belonging to clusters (n = 7) but also as singletons (n = 5). Nevertheless, the majority of moderate biofilm-producer strains were mainly found in clusters (n = 15/17).

The IR profiles 18 and 20 clustered mostly strains from patients not diagnosed with COVID-19, except for AC023, AC027 and AC035. Regarding cluster 4, the triplicate of the strains AC002 and AC016 showed twice due to the high similarity with the spectrum of AC005. This fact happens majorly due to the high clonality of *A. baumannii* strains, which suggests that these strains may belong to the same clone. These results corroborate those of the previous study where FTIR was able to discriminate all the *P. aeruginosa* strains and correctly clustered the strains related to co-infections with SARS-CoV-2 in one IR profile [29].

Other studies proved that IR Biotyper^®^ is an accurate tool for real-time epidemiological investigation of multidrug-resistant *A. baumannii* nosocomial outbreaks. In the study of Martak et al. [13], the FT-IR was able to differentiate 10 from 11 STs of 20 *A. baumannii* strains from outbreaks that occurred in hospitals from 11 French cities, reporting a clustering concordance of 0.915. Guerrero-Lozano [14] compared FT-IR with multilocus sequence typing (MLST) and pulsed-field gel electrophoresis (PFGE) techniques for typing 17 nosocomial *A. baumannii* strains isolated from a hospital. The authors observed that the FTIR results were very consistent with those from MLST, being able to differentiate between three STs (ST 1, ST 2 and ST80). Regarding PFGE, FTIR could differentiate 3/4 of the pulsotypes. Lombardo et al. [30] compared the FT-IR and PFGE for typing 24 *A. baumannii* strains isolated from ICU, and three different hospitals in the metropolitan area of Bologna, and concluded that FT-IR was fully confirmed by PFGE results. In the future, MLST or PFGE could be performed with the strains isolated from the present study to confirm whether it corroborates with FT-IR results.

The AC015 and AC024 strains, grouped in the IR 23 profile, were isolated from patients with COVID-19 who were hospitalized at ICU-2 and these strains were the only ones susceptible to GEN and SXT. Furthermore, IR profiles 5 and 6 clustered XDR strains that were isolated in the same month from patients with COVID-19. The IR 17 profile clustered the AC012 and AC013 strains, which were isolated from the same patient, on the same day but at different sources and formed a weak biofilm at 22.5 ± 2.5 °C. The strains AC001 and AC004 (Patient A), AC006 and AC007 (Patient E), AC018, AC022 and AC023 (Patient M), AC019 and AC020 (Patient N), AC021 and AC026 (Patient O), AC036 and AC038 (Patient Y), AC042 and AC046 (Patient AC) did not cluster with the strains that were isolated from the specific patient. This could indicate that possibly there were different sources of contamination in the hospital.

Infections caused by *A. baumannii* have become increasingly severe due to their antimicrobial resistance to commonly used antibiotics, such as β-lactams, aminoglycosides and even carbapenems [31]. Colistin is also reported as a valuable therapeutic option. Unfortunately, it was not possible to test this drug in the present study. According to Table A2, the highest level of resistance was observed for IMP, CIP and FOX with 97.9%, followed by 95.8% for TZP, CAZ, CEF and MEM. Elevated levels (87.3%) of resistance were also observed for AMI, AMS and CRO.

*A. baumannii* resistance to carbapenems is highlighted by the WHO as a formidable global challenge due to the limited treatment options, which leads to severe nosocomial infections [8]. Sulphonamides can be used as alternatives in the treatment of CARB [32]; however, in this study, 93.6% of the isolates were resistant to SXT, which restricts even more the therapeutic options available. These data corroborate the results provided in the study of Al-Tamimi et al. [33], where 49.9% of the *A. baumannii* strains were resistant to SXT.

TIG is a unique glycylcycline class of antibiotics and is considered a last resort antibiotics in the therapy of illness caused by MDR bacteria [34,35,36]. An increase in resistance rates to TIG among *Acinetobacter* spp. has been reported [35,36,37], with a high resistance rate of 66% found in Israel [37]. Although TIG had the lowest rate of resistance (21.3%) in the present study, the FDA [38] reported that there is an increased risk of mortality with tigecycline in comparison to other drugs. Furthermore, tigecycline is not recommended for use with children and teenagers under 18 years old [35].

The incidence of MDR and XDR *A. baumannii* strains represents a challenge in clinical treatment, and results in increasing rates of mortality around the world [39]. In the present study, most of the strains (91.5%) were classified as XDR and 6.4% of the strains were MDR (Figure 2), regardless of the COVID-19 co-infection, since only the AC015 strain, classified as non-MDR, was isolated from a patient with COVID-19. However, the AC015 strain still represents a challenge to public health since it was able to form moderately and strongly adherent biofilms at temperatures of 22.5 ± 2.5 °C and 37 ± 2 °C, respectively.

*A. baumannii* co-infection in patients with SARS-CoV-2 was widely reported during the pandemic due to its capacity to produce biofilms on medical devices used in endotracheal intubation [40]. *A. baumannii* biofilms contribute to its persistence in the hospital environment and consequently increase the risk of infection by this microorganism. In the present study, 57.4% of the strains were able to form moderately or strongly adherent biofilms at 37 °C in polystyrene surfaces (Figure 3). These data corroborate the results of the study of Yang et al. [9], where 32.5 and 45.4% of *A. baumannii* strains isolated from a teaching hospital formed moderate and strong biofilms, respectively. Vasconcellos et al. [25] also reported that 47.4% of *A. baumannii* strains were able to form strongly adherent biofilms in polystyrene surfaces.

*A. baumannii* biofilm formation also has an important role in antibiotic resistance and disinfectant tolerance, which makes the choice of the most effective treatment or cleaning protocols even more limited [11]. In this study, none of the disinfectants tested were able to reduce all the *A. baumannii* strains to non-adherent. Even though 70% alcohol is largely used for disinfection of hospital surfaces, in the present study, it was not efficient in the biofilm reduction (Figure 3). The most effective disinfectant and the only one to reduce some biofilms to non-adherent was sodium hypochlorite, which in the concentrations of 0.5% and 1.0% reduced 29.6 and 85.12% of the strains, respectively, to non-adherent (Figure 3). However, it cannot be used on surfaces that are sensitive to corrosion. For that reason, other sanitizers and disinfectants and time exposure must be evaluated.

## 5. Conclusions

In conclusion, VITEK MS^®^, MALDI Biotyper^®^ and 16S rRNA gene sequencing accurately identified all *A. baumannii* strains. FTIR was considered a useful tool for *A. baumannii* typing since it was able to differentiate the strains from patients with and without COVID-19. Most of the isolates were intermediate resistant or resistant to the antibiotics evaluated, while TIG was demonstrated to be the best treatment option for *A. baumannii* with the lowest rate of resistance. Almost all strains (n = 43) were classified as XDR, while three strains were MDR and only one strain was classified as non-MDR. Over half of the strains (n = 27) were able to form moderately or strongly adherent biofilm on stainless steel surfaces. It was observed that *A. baumannii* strains already possess these virulent characteristics regardless of COVID-19.

## Figures and Tables

**Table 1:** Details of *Acinetobacter baumannii* strains (n = 47) isolated in a Brazilian hospital during the COVID-19 pandemic.

Strain (CBAS ^a^ ID)	Source	Patient ID	Hospital Department	COVID-19 Co-Infection	Isolation Date	VITEK^®^ 2 Bionumber (Profile)	MALDI-TOF MS ^b^	
							VITEK MS^®^ (%)	MALDI Biotyper^®^ (Score)
AC001/21 (936)	Tracheal swab	A	ICU ^c^-1	no	21 June	0241010103500250 (I)	*A. baumannii* (98.4)	*A. baumannii* (2.43)
AC002/21(937)	Tracheal swab	B	ICU-2	yes	21 June	0201010103500210 (II)	*A. baumannii* (99.9)	*A. baumannii* (2.33)
AC003/21 (938)	Tracheal swab	C	ICU-2	yes	21 June	0241010103500352 (III)	*A. baumannii* (99.9)	*A. baumannii* (2.43)
AC004/21 (939)	Tracheal swab	A	ICU-1	no	21 June	0241010103500210 (IV)	*A. baumannii* (95.0)	*A. baumannii* (2.31)
AC005/21 (940)	Blood	D	ICU-2	yes	21 June	0201010103500352 (V)	*A. baumannii* (99.9)	*A. baumannii* (2.36)
AC006/21 (941)	Blood	E	ICU-2	yes	21 June	0201010103500312 (VI)	*A. baumannii* (99.9)	*A. baumannii* (2.28)
AC007/21 (942)	Blood	E	ICU-2	yes	21 June	0241010103500352 (III)	*A. baumannii* (99.9)	*A. baumannii* (2.04)
AC008/21 (943)	Blood	E	ICU-2	yes	21 June	0241010103500310 (VII)	*A. baumannii* (81.7)	*A. baumannii* (2.25)
AC009/21 (944)	Urine	F	ICU-2	yes	21 July	0201010103500210 (II)	*A. baumannii* (99.9)	*A. baumannii* (2.33)
AC010/21 (945)	Tracheal secretion	G	ICU-2	Yes	21 July	0201010103500210 (II)	*A. baumannii* (99.9)	*A. baumannii* (2.38)
AC012/21 (946)	Urine	H	ICU-2	Yes	21 July	0241010103500310 (VII)	*A. baumannii* (99.9)	*A. baumannii* (2.21)
AC013/21 (947)	Tracheal secretion	H	ICU-2	Yes	21 July	0201010103500352 (V)	*A. baumannii* (99.9)	*A. baumannii* (2.22)
AC014/21 (948)	Blood	E	ICU-2	Yes	21 July	0241010003500312 (VIII)	*A. baumannii* (99.9)	*A. baumannii* (2.32)
AC015/21 (949)	Blood	I	ICU-2	Yes	21 July	0201010103500312 (VI)	*A. baumannii* (97.0)	*A. baumannii* (2.29)
AC016/21 (950)	Blood	J	ICU-1	No	21 July	0241010103500310 (VII)	*A. baumannii* (99.9)	*A. baumannii* (2.26)
AC017/21 (951)	Blood	L	ICU-2	Yes	21 September	0241010103500210 (IV)	*A. baumannii* (99.9)	*A. baumannii* (2.15)
AC018/22 (952)	Rectal swab	M	ICU-1	No	22 January	0241010103500250 (I)	*A. baumannii* (81.3)	*A. baumannii* (2.25)
AC019/22 (953)	Rectal swab	N	ICU-1	No	22 January	0241010003500210 (IX)	*A. baumannii* (83.0)	*A. baumannii* (2.37)
AC020/22 (954)	Tracheal secretion	N	ICU-1	No	22 February	0241010103500212 (X)	*A. baumannii* (99.4)	*A. baumannii* (2.23)
AC021/22 (955)	Tracheal secretion	O	ICU-2	Yes	22 February	0241010103500352 (III)	*A. baumannii* (99.9)	*A. baumannii* (2.33)
AC022/22 (956)	Urine	M	ICU-1	No	22 January	0241010103500210 (IV)	*A. baumannii* (99.9)	*A. baumannii* (2.36)
AC023/22 (957)	Tracheal secretion	M	ICU-2	Yes	22 February	0241010103500210 (IV)	*A. baumannii* (99.9)	*A. baumannii* (2.02)
AC024/22 (958)	Tracheal secretion	P	ICU-2	Yes	22 February	0241010103500312 (XI)	*A. baumannii* (99.9)	*A. baumannii* (2.36)
AC025/22 (959)	Tracheal secretion	Q	ICU-2	Yes	22 February	0241010003500310 (XII)	*A. baumannii* (99.9)	*A. baumannii* (2.32)
AC026/22 (960)	Blood	O	ICU-2	Yes	22 February	0241010103500310 (VII)	*A. baumannii* (98.0)	*A. baumannii* (2.41)
AC027/22 (961)	Blood	R	ICU-2	Yes	22 February	0201010103500210 (II)	*A. baumannii* (99.9)	*A. baumannii* (2.00)
AC028/22 (962)	Tracheal secretion	S	ICU-1	no	22 February	0201010003500210 (XIII)	*A. baumannii* (99.9)	*A. baumannii* (2.31)
AC029/22 (963)	Tracheal secretion	T	ICU-1	no	22 February	0241010103500310 (VII)	*A. baumannii* (99.9)	*A. baumannii* (2.24)
AC030/22 (964)	Tracheal secretion	U	Medical clinic-1	no	22 February	0241010003500310 (XII)	*A. baumannii* (99.9)	*A. baumannii* (2.41)
AC031/22 (965)	Blood	V	ICU-1	no	22 February	0241010103500210 (IV)	*A. baumannii* (88.8)	*A. baumannii* (2.29)
AC032/22 (966)	Rectal swab	X	ICU-2	yes	22 February	0243051103500352 (XIV)	*A. baumannii* (99.5)	*A. baumannii* (2.46)
AC033/22 (967)	Rectal swab	Z	ICU-2	yes	22 February	0201010003500210 (XIII)	*A. baumannii* (99.9)	*A. baumannii* (2.13)
AC034/22 (968)	Tracheal secretion	S	ICU-1	no	22 February	0241010103500210 (IV)	*A. baumannii* (99.9)	*A. baumannii* (2.44)
AC035/22 (969)	Rectal swab	W	ICU-2	yes	22 February	0241010003500210 (IX)	*A. baumannii* (96.2)	*A. baumannii* (2.20)
AC036/22 (970)	Urine	Y	ICU-2	yes	22 February	0201010001500210 (XV)	*A. baumannii* (99.9)	*A. baumannii* (2.36)
AC037/22 (971)	Tracheal secretion	K	ICU-1	no	22 March	0241010103500352 (III)	*A. baumannii* (96.4)	*A. baumannii* (2.40)
AC038/22 (672)	Tracheal secretion	Y	ICU-2	yes	22 March	0241010103500310 (VII)	*A. baumannii* (99.9)	*A. baumannii* (2.45)
AC039/22 (973)	Tracheal secretion	AB	ICU-1	no	22 February	0241010103500310 (VII)	*A. baumannii* (95.4)	*A. baumannii* (2.07)
AC040/22 (974)	Tracheal secretion	S	ICU-1	no	22 February	0241010103500210 (IV)	*A. baumannii* (91.9)	*A. baumannii* (2.01)
AC041/22 (975)	Urine	Z	ICU-2	yes	22 March	0241010103500210 (IV)	*A. baumannii* (88.8)	*A. baumannii* (2.49)
AC042/22 (976)	Rectal swab	AC	ICU-2	yes	22 March	0241010003500210 (IX)	*A. baumannii* (99.9)	*A. baumannii* (2.17)
AC043/22 (977)	Rectal swab	AD	ICU-2	yes	22 March	0241010103500310 (VII)	*A. baumannii* (92.0)	*A. baumannii* (2.25)
AC044/22 (978)	Rectal swab	AE	ICU-1	no	22 March	0201010003500210 (XIII)	*A. baumannii* (99.9)	*A. baumannii* (2.44)
AC045/22 (979)	Rectal swab	AF	ICU-1	no	22 March	0201010103500312 (VI)	*A. baumannii* (91.8)	*A. baumannii* (2.30)
AC046/22 (1030)	Tracheal secretion	AC	ICU-2	yes	22 March	0201010103500210 (II)	*A. baumannii* (92.0)	*A. baumannii* (2.20)
AC047.22 (980)	Urine	AG	Medical clinic-2	no	22 March	0241411103500353 (XVI)	*A. baumannii* (99.9)	*A. baumannii* (2.25)
AC048.22 (981)	Rectal swab	AH	ICU-2	no	22 March	0241010103500310 (VII)	*A. baumannii* (99.9)	*A. baumannii* (2.05)

^a^—Coleção de Bactérias do Ambiente e Saúde/Fundação Oswaldo Cruz; ^b^—matrix-assisted laser desorption ionization–time of flight mass spectrometry performed; ^c^—intensive care unit.

## Data Availability

The data underlying this article are available in the article.

## Appendix A

### Appendix A.1

**Table A1:** 16S rDNA sequencing analysis of *Acinetobacter baumannii* strains (n = 47).

Strain ID	NCBI ^a^ Access Number	Base Pair Length (nt)	Identification (%)
AC001/21	OQ955834	728	*A. baumannii* (99.59)
AC002/21	OQ955835	842	*A. baumannii* (99.88)
AC003/21	OQ955836	860	*A. baumannii* (99.88)
AC004/21	OQ955837	704	*A. baumannii* (97.28)
AC005/21	OQ955838	851	*A. baumannii* (99.88)
AC006/21	OQ955839	974	*A. baumannii* (99.90)
AC007/21	OQ955840	858	*A. baumannii* (99.88)
AC008/21	OQ955841	1.030	*A. baumannii* (99.90)
AC009/21	OQ955842	984	*A. baumannii* (99.90)
AC010/21	OQ955843	940	*A. baumannii* (99.90)
AC012/21	OQ955844	874	*A. baumannii* (99.88)
AC013/21	OQ955845	980	*A. baumannii* (99.90)
AC014/21	OQ955846	943	*A. baumannii* (99.89)
AC015/21	OQ955847	922	*A. baumannii* (99.89)
AC016/21	OQ955848	861	*A. baumannii* (99.88)
AC017/21	OQ955849	986	*A. baumannii* (99.90)
AC018/22	OQ955850	1.036	*A. baumannii* (99.90)
AC019/22	OQ955851	945	*A. baumannii* (99.89)
AC020/22	OQ955852	984	*A. baumannii* (99.89)
AC021/22	OQ955853	860	*A. baumannii* (99.88)
AC022/22	OQ955854	884	*A. baumannii* (99.77)
AC023/22	OQ955855	845	*A. baumannii* (99.88)
AC024/22	OQ968344	1.036	*A. baumannii* (99.71)
AC025/22	OQ968345	1.034	*A. baumannii* (99.90)
AC026.22	OQ968346	1.037	*A. baumannii* (99.90)
AC027/22	OQ968347	873	*A. baumannii* (99.88)
AC028/22	OQ968348	945	*A. baumannii* (99.68)
AC029/22	OQ968349	1.034	*A. baumannii* (99.90)
AC030/22	OQ968350	1.043	*A. baumannii* (99.61)
AC031/22	OQ968351	1.043	*A. baumannii* (99.90)
AC032/22	OQ968352	1.033	*A. baumannii* (99.90)
AC033/22	OQ970134	1.031	*A. baumannii* (99.90)
AC034/22	OQ970135	1.034	*A. baumannii* (99.90)
AC035/22	OQ970136	1.042	*A. baumannii* (99.90)
AC036/22	OQ970137	453	*A. baumannii* (99.77)
AC037/22	OQ970138	1.031	*A. baumannii* (99.90)
AC038/22	OQ970139	1.032	*A. baumannii* (99.90)
AC039/22	OQ970140	1.033	*A. baumannii* (99.90)
AC040/22	OQ970141	1.032	*A. baumannii* (99.90)
AC041/22	OQ970142	1.020	*A. baumannii* (90.90)
AC042/22	OQ970143	1.035	*A. baumannii* (90.90)
AC043/22	OQ970144	1.034	*A. baumannii* (90.90)
AC044/22	OQ970145	1.003	*A. baumannii* (90.90)
AC045/22	OQ970146	1.034	*A. baumannii* (90.90)
AC046/22	PP373676	1.306	*A. baumannii* (90.90)
AC047/22	OQ970147	1.006	*A. baumannii* (100)
AC048/22	OQ970148	1.003	*A. baumannii* (100)

^a^ National Center for Biotechnology Information.

### Appendix A.2

**Table A2:** Antimicrobial susceptibility profile of *Acinetobacter baumannii* strains (n = 47).

Strains	AMS	TZP	FOX	CAZ	CRO	CEF	IPM	MEM	GEN	CIP	TIG	SXT	AMI	Magiorakos et al. [1] Classification
AC001.21	I	R	R	R	R	R	R	R	R	R	S	R	R	XDR
AC002.21	R	R	R	R	R	R	R	R	R	R	S	R	R	XDR
AC003.21	R	R	R	R	R	R	R	R	R	R	S	R	R	XDR
AC004.21	R	R	R	R	R	R	R	R	R	R	S	R	R	XDR
AC005.21	R	R	R	R	R	R	R	R	R	R	S	R	S	XDR
AC006.21	R	R	R	R	I	R	R	R	R	R	R	R	R	XDR
AC007.21	R	R	R	R	I	R	R	R	R	R	R	R	R	XDR
AC008.21	R	R	R	R	I	R	R	R	R	R	R	R	R	XDR
AC010.21	R	R	R	R	R	R	R	R	R	R	S	R	R	XDR
AC012.21	R	R	R	R	R	R	R	R	R	R	S	R	R	XDR
AC013.21	R	R	R	R	R	R	R	R	R	R	I	R	R	XDR
AC014.21	S	R	R	R	I	R	R	R	R	R	R	R	R	XDR
AC015.21	S	S	R	S	I	S	S	S	S	I	S	S	S	N-MDR
AC016.21	R	R	R	R	R	R	R	R	R	R	S	R	R	XDR
AC017.21	R	R	R	R	R	R	R	R	R	R	I	R	R	XDR
AC018.22	R	R	R	R	R	R	R	R	R	R	I	R	S	XDR
AC019.22	R	R	R	R	R	R	R	R	R	R	I	R	R	XDR
AC020.22	R	R	R	R	R	R	R	R	R	R	I	R	R	XDR
AC021.22	R	R	R	R	R	R	R	R	R	R	I	R	R	XDR
AC022.22	S	R	R	R	R	R	R	R	R	R	S	R	R	XDR
AC023.22	R	R	R	R	R	R	R	R	R	R	S	R	R	XDR
AC024.22	R	R	R	R	R	R	R	R	S	R	I	S	R	MDR
AC025.22	R	R	R	R	R	R	R	R	S	R	S	R	S	MDR
AC026.22	R	R	R	R	R	R	R	R	R	R	I	R	R	XDR
AC027.22	R	R	R	R	R	R	R	R	R	R	S	R	R	XDR
AC028.22	R	R	R	R	R	R	R	R	R	R	I	R	R	XDR
AC029.22	R	R	R	R	R	R	R	R	R	R	S	R	R	XDR
AC030.22	R	R	R	R	R	R	R	R	R	R	R	S	S	XDR
AC031.22	R	R	R	R	R	R	R	R	R	R	I	R	R	XDR
AC032.22	R	R	R	R	R	R	R	R	R	R	I	R	R	XDR
AC033.22	R	R	R	R	R	R	R	R	R	R	R	R	R	XDR
AC034.22	R	R	R	R	R	R	R	R	R	R	R	R	R	XDR
AC035.22	R	R	R	R	R	R	R	R	R	R	I	R	R	XDR
AC036.22	R	R	R	R	R	R	R	R	R	R	S	R	R	XDR
AC037.22	R	R	R	R	R	R	R	R	R	R	I	R	R	XDR
AC038.22	R	R	R	R	R	R	R	R	R	R	I	R	R	XDR
AC039.22	R	R	R	R	R	R	R	R	R	R	R	R	R	XDR
AC040.22	R	R	R	R	R	R	R	R	R	R	R	R	R	XDR
AC041.22	I	R	R	R	R	R	R	R	R	R	S	R	R	XDR
AC042.22	R	R	R	R	R	R	R	R	R	R	R	R	R	XDR
AC043.22	R	R	R	R	R	R	R	R	R	R	S	R	R	XDR
AC044.22	R	R	R	R	R	R	R	R	R	R	I	R	R	XDR
AC045.22	R	R	R	R	R	R	R	R	R	R	I	R	R	XDR
AC046.22	R	R	R	R	R	R	R	R	R	R	S	R	R	XDR
AC047.22	S	S	I	S	S	S	R	S	R	R	S	R	S	MDR
AC048.22	R	R	R	R	R	R	R	R	R	R	I	R	R	XDR

R: resistant; I: intermediate resistant; S: susceptible; MDR: multidrug-resistant; XDR: extensively drug-resistant; N-MDR: non-multidrug-resistant; meropenem (MEM); imipenem (IPM); sulfamethoxazole–trimethoprim (SXT); piperacillin–tazobactam (TZP); amikacin (AMI); gentamicin (GEN); ciprofloxacin (CIP); ceftazidime (CAZ); cefoxitin (FOX); cefepime (CEF); ceftriaxone (CRO); tigecycline (TIG); ampicillin–sulbactam (AMS).
